# Entomological efficacy of durable wall lining with reduced wall surface coverage for strengthening visceral leishmaniasis vector control in Bangladesh, India and Nepal

**DOI:** 10.1186/s12879-016-1881-8

**Published:** 2016-10-06

**Authors:** M. Mamun Huda, Vijay Kumar, Murari Lal Das, Debashis Ghosh, Jyoti Priyanka, Pradeep Das, Abdul Alim, Greg Matlashewski, Axel Kroeger, Eduardo Alfonso-Sierra, Dinesh Mondal

**Affiliations:** 1 NCSD and Parasitology Laboratory, International Centre For Diarrhoeal Disease Research, Bangladesh (icddr,b), 68 Shaheed Taj Uddin Ahmed Sarani, Mohakhali, Dhaka, 1212 Bangladesh; 2 Rajendra Memorial Research Institute of Medical Sciences, Patna, India; 3 Entomology laboratory, Department of Microbiology, BP Koirala Institute of Health Sciences, Dharan, Nepal; 4 Department of Microbiology and Immunology, McGill University, Montreal, Canada; 5 UNICEF/UNDP/World Bank/WHO Special Programme for Research and Training in Tropical Diseases (WHO/TDR), Geneva, Switzerland; 6 Centre for Medicine and Society, University Medical Centre Freiburg, Freiburg, Germany

**Keywords:** Visceral leishmaniasis, Kala-azar, Sand fly, Vector control, Durable wall lining, Bangladesh, India, Nepal, TDR

## Abstract

**Background:**

New methods for controlling sand fly are highly desired by the Visceral Leishmaniasis (VL) elimination program of Bangladesh, India and Nepal for its consolidation and maintenance phases. To support the program we investigated safety, efficacy and cost of Durable Wall Lining to control sand fly.

**Methods:**

This multicentre randomized controlled study in Bangladesh, India and Nepal included randomized two intervention clusters and one control cluster. Each cluster had 50 households except full wall surface coverage (DWL-FWSC) cluster in Nepal which had 46 households. Ten of 50 households were randomly selected for entomological activities ﻿except India where it was 6 households. Interventions were DWL-FWSC and reduced wall surface coverage (DWL-RWSC) with DWL which covers 1.8 m and 1.5 m height from floor respectively. Efficacy was measured by reduction in sand fly density by intervention and sand fly mortality assessment by the WHO cone bioassay test at 1 month after intervention. Trained field research assistants interviewed household heads for socio-demographic information, knowledge and practice about VL, vector control, and for their experience following the intervention. Cost data was collected using cost data collection tool which was designed for this study. Statistical analysis included difference-in-differences estimate, bivariate analysis, Poisson regression model and incremental cost-efficacy ratio calculation.

**Results:**

Mean sand fly density reduction by DWL-FWSC and DWL-RWSC was respectively −4.96 (95 % CI, −4.54, −5.38) and −5.38 (95 % CI, −4.89, −5.88). The sand fly density reduction attributed by both the interventions were statistically significant after adjusting for covariates (IRR = 0.277, *p* < 0.001 for DWL-RWSC and IRR = 0.371, *p* < 0.001 for DWL-FWSC). The efficacy of DWL-RWSC and DWL-FWSC on sand fly density reduction was statistically comparable (*p* = 0.214). The acceptability of both interventions was high. Transient burning sensations, flash on face and itching were most common adverse events and were observed mostly in Indian site. There was no serious adverse event. DWL-RWSC is cost-saving compared to DWL-FWSC. The incremental cost-efficacy ratio was −6.36, where DWL-RWSC dominates DWL-FWSC.

**Conclusions:**

DWL-RWSC intervention is safe, efficacious, cost-saving and cost-effective in reducing indoor sand fly density. The VL elimination program in the Indian sub-continent may consider DWL-RWSC for sand fly control for its consolidation and maintenance phases.

**Electronic supplementary material:**

The online version of this article (doi:10.1186/s12879-016-1881-8) contains supplementary material, which is available to authorized users.

## Background

Visceral leishmaniasis (VL) continues to be a public health problem in the Indian sub-continent over decades. VL is known as kala-azar in the Indian sub-continent. The estimated annual incidence of VL in India, Bangladesh and Nepal is about 314,000 [1]. VL affects poor people in the rural areas of these countries causing substantial economic loss in these countries as well affected individual families [1–3]. *Leishmania donovani* is the only parasite causing VL in the sub-continent and it is transmitted by the infected sand fly *Phlebotomus argentipes*. The first reported outbreak of VL was in 1824 in a territory of Bangladesh when 75,000 people died [4].

Fortunately the burden of VL is now going down in the Indian sub-continent [5, 6]. In 2005 the Governments of India, Bangladesh and Nepal committed to a VL elimination program to sustainably bring down the number of cases to less that 1 per 10,000 people at the district/upazila (sub-district) level by 2015 [7]. Nepal achieved the elimination target and Bangladesh is very close to the achievement [8]. Early case detection and proper management and indoor residual spraying with insecticides (IRS) for sand fly control were the pillars of success in the attack phase of the elimination program [5, 6, 8]. In the subsequent consolidation and maintenance phases of the program the Government of these countries may be reluctant to use IRS for controlling sand fly because of its cost and in a situation when VL burden has substantially went down to few hundreds. So, new cost-effective and durable vector control methods are needed.

Other than IRS currently available methods for vector control are Long Lasting Insecticide Treated bed-net (either commercial or conversion of existing household bed-net into LLIN by their impregnation with slow release insecticide tablets), and durable wall lining (DWL) [9–12]. Durable Wall Lining (DWL, ZeroVectorTM, Vestergaard, Switzerland) contains a thin polyethylene material impregnated with deltamethrin in a concentration of Deltamethrin 170 mg a.i./m^2^. In our previous study we showed that among these methods the DWL was the most effective but costly intervention to control sand fly in Bangladesh, India and Nepal [13]. In that study the intervened indoor household walls were covered by DWL up to 1.8 m in height from the floor (refer to as full wall surface coverage, DWL-FWSC) and 72 % reduction of sand fly density at month after intervention was achieved [13]. It is believed that sand fly usually rests in the lower part of indoor walls. So installation DWL in 1.5 m in height from floor (refer to as DWL-RWSC) should be sufficient to obtain the same entomological efficacy against sand fly as with full wall surface coverage with 1.8 m DWL.

In this study we aimed to compare the entomological efficacy of reduced wall surface coverage with 1.5 m DWL (DWL-RWSC) versus full wall surface coverage with 1.8 m DWL (DWL-FWSC) against sand fly.

## Methods

### Study sites, design and population

This multi-center study was a cluster randomized controlled design and was conducted in Bangladesh, India and Nepal from March 2014 to December 2014. Each study site had two intervention clusters and one control cluster. Intervention clusters included one cluster with DWL-FWSC and one cluster with DWL-RWSC. In India and Bangladesh three VL endemic villages were selected and a cluster of 50 households were included in each village. In Nepal, three clusters were in one VL endemic village where control and DWL-RWSC clusters had 50 households and DWL-FWSC cluster had 46 households (Table 1).

**Table 1:** Study Profile

	Bangladesh	India	Nepal	Overall/ Pooled
No. of District	1	1	1	3
No. of PHCs/Upazilas/VDCs	1	1	1	3
No. of Villages	3	3	1	7
No. of Cluster	3	3	3	9
- DWL-RWSC cluster	1	1	1	3
- DWL-FWSC cluster	1	1	1	3
- Control cluster	1	1	1	3
Total Household	150	150	146	446
- DWL-RWSC cluster	50	50	50	150
- DWL-FWSC cluster	50	50	46	146
- Control cluster	50	50	50	150
Total Population	630	725	736	2091
- DWL-RWSC cluster	217	232	239	688
- DWL-FWSC cluster	208	260	237	705
- Control cluster	205	233	260	698

*Abbreviations*: *PHC* Primary Health Centre, *Upazila* sub-district, *VDC* village development committee, *DWL* Durable Wall Lining, *DWL*-*RWSC* wall surface coverage with DWL up to 1.5 m in height from floor, *DWL*-*FWSC* wall surface coverage with DWL up to 1.8 m in height from floor

### Sample size estimation for entomological activities

We assumed that the effect on sand fly density reduction would be the same in each of the interventions. To calculate sample size, we expected 60 % reduction of mean sand fly (female *P. argentipes*) count per household [13] by the DWL intervention following our previous study results of 56 % (95 % CI, 47 %–70 %) reduction of female *P. argentipes* sand fly count at 1-month follow-up [13]. Our previous study also showed that pooled mean density of female *P. argentipes* sand fly was 5.35 count per household [13]. We observed that the variation in sand fly count data at house level was also high. Therefore, considering mean female *P. argentipes* sand fly count per household about 5.35 (SD = 3.0) and 3.21 before and after intervention respectively, a study power of 80 % and 5 % level of significance, the minimum pooled number of household for entomological measurements was 23 per arm. So in total the required minimum number was 23 × 3 = 69. We had 30 households in each study site (10 households per arm) except I﻿ndia where it was 18 households﻿ (6 households per arm)﻿ and in total (30 × 2) +18 = 78 households.

### Measurement of efficacy of intervention

**Fig. 1:**
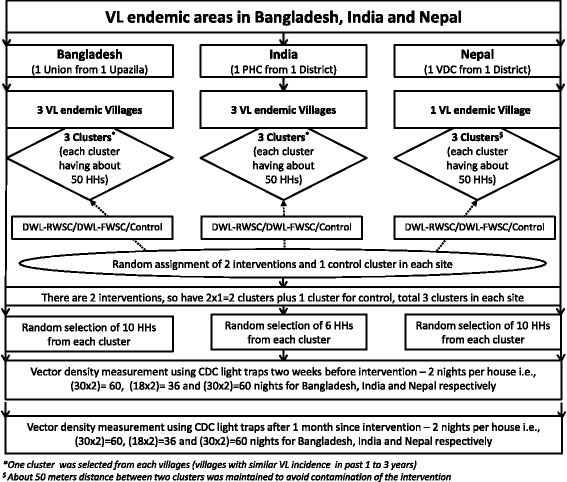
Study design

Efficacy of the intervention was defined by the reduction of mean female *P. argentipes* sand fly count after intervention in intervention clusters compare to control cluster. Ten households in each cluster of each study site were randomly assigned for entomological activities for measurement of intervention efficacy except India where it was 6 households in each cluster (Fig. 1). Abbot’s corrected sand fly mortality after exposure to DWL-RWSC and DWL-FWSC using a WHO cone bio-assay test was another indicator for intervention efficacy assessment.

### Sand fly density measurement

Trained field research assistants and health workers collected sand flies on 2 consecutive nights from 6 pm to 6 am using CDC light traps as described earlier [12]. Sand fly collection had been done in March–May in Bangladesh and April–June in India and Nepal which complied with peak sand fly seasonality. As mentioned above, sand fly density was defined by mean number of female *P. argentipes* per house. An entomologist dissected collected sand flies and identified sand fly species by morphological examination following standard methodology [14]. Entomological activities were carried out before intervention and at 1 month after intervention.

### Household interview

Trained field research assistants interviewed household head for collection of socio-economic information, his/her knowledge about kala-azar, vector control practices and for their experiences / opinion about the intervention he / she had in their houses. Interviews were conducted 6 weeks after intervention.

### Statistical analysis

Descriptive analysis was performed to determine the average sand fly density among different interventions and control groups at baseline and at 1 month follow-up after the intervention. Mean female *P. argentipes* sand fly counts between control and intervention areas were compared using a Mann Whitney *U* test. The intervention effect was estimated using the following formula which includes the difference of the differences and should be zero if there is no intervention effect and negative if there is a larger reduction in the intervention clusters compared to the control arm.

Effect of intervention: (B-A)–(D-C): A = baseline value for the intervention arm; B = post-intervention value for the intervention arm; C = baseline value for the control arm; D = post-intervention value for the control arm.

For the overall efficacy analysis, data from the three study sites were pooled, including the baseline survey and 1 month follow-up to determine the effect of the intervention. It was found that the Poisson distribution fitted the data and all analyses were performed under that assumption. Generalized estimating equation (GEE) modeling was used to adjust the correlation in data due to longitudinal/repeated measurements in cluster sampling. In the model, an interaction term of being in the intervention arm at follow-up was included in order to estimate the effect of the intervention. Technically the regression model has the following structure: Count = Intercept + a*Treatment (1 for intervention and 0 for control) + b*Time (1 for follow up and 0 for baseline) + c*Interaction (1 for intervention group at follow up and 0 for otherwise) + error. The c-coefficient assesses intervention effect.

Two types of model were fitted, one for DWL-RWSC vs Control group and another for DWL-FWSC vs Control group. Finally these two types of models were compared using the Chow test to determine differences between efficacies of DWL-RWSC and DWL-FWSC interventions. In the tables, only the IRR (Incidence Rate Ratio) corresponding to c-coefficient and its *p*-value are given. Significances are given at the 5 % level and 95 % confidence intervals. In this report, we will focus on the pooled analysis of the three study sites in order to draw general conclusions but will also present site-specific results. The main outcome variable was 'Female *P. argentipes* sand flies count' per household before and 1 month after intervention. The variables which varied between intervention and control areas with *p*-values less than or equal 0.20 are considered as covariates for the full model to identify the adjusted effect of the interventions. All the analysis was perform by using STATA 10.1. Cost data were collected using cost data collection tool which was designed for this study. Average intervention cost per household was calculated. Cost of the both interventions was compared using incremental cost efficacy ratio [14]. Probabilistic sensitivity analysis was conducted assuming a normal distribution, using mean and standard deviation of the efficacy estimates and the cost per household and 15 % coefficient of variation for costs estimates.

## Results

### Study clusters’ characteristics

Households in the study clusters (pooled) in the three countries did not differ regarding household head education, profession, family size and asset scores (Table 2). However, households with mud floors in control group was significantly more common compare to households in the DWL-FWSC cluster (*p* = 0.051) (Table 2). This variation was due to the difference among houses with mud floor in clusters in Nepal (see Additional file 1: Table S1). Household asset score significantly differ between clusters in Bangladesh and this was not the case in Nepal and India; however in the pooled data asset scores were similar among different clusters (Additional file 1: Table S1 and Table 1). Household head’s knowledge about kala-azar did not differ among the clusters. Vector control practices were limited to the use of bed-nets, smoked/dhup and mosquito coil and were similar across the clusters. Households in the control clusters did not have indoor residual spraying (IRS) by the national program in the previous 6 months, however 38 and 3.8 % households respectively in DWL-FWSC and DWL-RWSC clusters did (*p* = 0.001). This variation was due to significant variation between clusters in Bangladesh site where all households in DWL-FWSC cluster had IRS in the previous 6 months (Additional file 1: Table S1). Ten percent of households with DWL-RWSC intervention in Nepal also had IRS in the previous 6 months.

**Table 2:** Bivariate analysis of household related variables in the study area (Pooled data)

	Control cluster, % (*n*)	Intervention cluster				Total, % (*n*)
		DWL-RWSC, % (*n*)	*p*-value	DWL-FWSC, % (*n*)	*p*-value	
Overall (pooled)	*N* = 26	*N* = 26		*N* = 26		*N* = 78
Illiterate household head	38.5 (10)	42.3 (11)	1.000	19.2 (5)	0.220	33.3 (26)
Labor household head	73.1 (19)	73.1 (19)	1.000	50.0 (13)	0.153	65.4 (51)
Family size > =5	57.7 (15)	38.5 (10)	0.267	61.5 (16)	1.000	52.6 (41)
Bed-rooms <2	46.2 (12)	53.8 (14)	0.782	42.3 (11)	1.000	47.4 (37)
Family members slept at Varanda during the hot season	65.4 (17)	42.3 (11)	0.164	88.5 (23)	0.097	65.4 (51)
Having cattle shed	65.4 (17)	69.2 (18)	1.000	69.2 (18)	1.000	67.9 (53)
Housing materials:						
- Mud wall	84.6 (22)	92.3 (24)	0.668	65.4 (17)	0.199	80.8 (63)
- Mud floor	100 (26)	92.3 (24)	0.490	80.8 (21)	0.051	91.0 (71)
HH asset score						
- Low	61.5 (16)	50.0 (13)	0.577	38.5 (10)	0.165	50.0 (39)
- Medium	0.0 (0)	0.0 (0)		0.0 (0)		0.0 (0)
- High	38.5 (10)	50.0 (13)		61.5 (16)		50.0 (39)
Crack in wall	65.4 (17)	65.4 (17)	1.000	53.8 (14)	0.572	61.5 (48)
Damp floor	0.0 (0)	3.8 (1)	1.000	0.0 (0)	–	1.3 (1)
HH head aware about VL	96.2 (25)	96.2 (25)	1.000	80.8 (21)	0.191	91.0 (71)
HH head aware about VL vector	26.9 (7)	0.0 (0)	0.010	19.2 (5)	0.743	15.4 (12)
Having bed-net in house	80.8 (21)	80.8 (21)	1.000	88.5 (23)	0.703	83.3 (65)
# bed-net <2 in house	38.5 (10)	42.3 (11)	1.000	11.5 (3)	0.052	30.8 (24)
Regular use of bed-net	38.5 (10)	53.8 (14)	0.404	34.6 (9)	1.000	42.3 (33)
Other insecticides use:						
- Mosquito coil	19.2 (5)	3.8 (1)	0.191	11.5 (3)	0.703	11.5 (9)
- Repellents	0.0 (0)	0.0 (0)	–	0.0 (0)	–	0.0 (0)
- Spray	3.8 (1)	0.0 (0)	1.000	0.0 (0)	1.000	1.3 (1)
- Smoke/dhup	26.9 (7)	23.1 (6)	1.000	23.1 (6)	1.000	24.4 (19)
- Others	0.0 (0)	0.0 (0)	–	0.0 (0)	–	0.0 (0)
House sprayed with insecticide (IRS) within last 6 months	0.0 (0)	3.8 (1)	1.000	38.5 (10)	0.001	14.1 (11)

*Abbreviations*: *HH* household, *IRS* indoor residual spraying with insecticides, *DWL*-*RWSC* wall surface coverage with DWL up to 1.5 m in height from floor, *DWL*-*FWSC* wall surface coverage with DWL up to 1.8 m in height from floor

### Intervention efficacy

#### Crude intervention effect on sand fly density

The mean sand fly before interventions in DWL-RWSC clusters did not differ significantly with mean sand fly density in control clusters and these findings were the same across the sites (Table 3). However the sand fly density was significantly less in DWL-FWSC clusters compare to control clusters in Bangladesh and India (Table 3). At 1 month after intervention, the sand fly density in DWL-RWSC clusters, DWL-FWSC clusters and in control clusters were respectively 2.00 (95 % CI, 1.49–2.62), 2.92 (95 % CI, 2.30–3.66) and 8.65 (95 % CI, 7.56–9.86). Both intervention clusters differed significantly from the control clusters (Table 3).

**Table 3:** Female *P. arentipes* sand fly per household in pooled as well as site specific data and their comparison between interventions and control arm at baseline and follow-up time

Survey	Mean (95 % CI) Female *P. argentipes* sand fly per household			*p*-value for test of differences	
	DWL-RWSC	DWL-FWSC	Control	IDWL-RWSC vs Control	IDWL-FWSC vs Control
Pooled					
- Baseline	6.65 (5.70, 7.72)	7.15 (6.16, 8.26)	7.92 (6.88, 9.08)	0.6792	0.0934
- End line	2.00 (1.49, 2.62)	2.92 (2.30, 3.66)	8.65 (7.56, 9.86)	<0.0001	<0.0001
- Difference in differences compared to control arm^a^	−5.38 (−4.89, −5.88)	−4.96 (−4.54, −5.38)	–		
Site specific					
Bangladesh					
- Baseline	7.2 (5.63, 9.07)	4.1 (2.94, 5.56)	9.8 (7.96, 11.94)	0.9394	0.0245
- End line	2.9 (1.94, 4.16)	1.5 (0.84, 2.47)	8.3 (6.61, 10.29)	0.0566	0.0061
- Difference in differences compared to control arm ^a^	−2.8 (−2.34, −3.26)	−1.1 (−0.75, −1.44)	–		
India					
- Baseline	9.0 (6.76, 11.74)	4.0 (2.56, 5.95)	8.67 (6.47, 11.37)	0.8661	0.0056
- End line	0.33 (0.04, 1.20)	0.17 (0.00, 0.93)	4.67 (3.10, 6.74)	0.0035	0.0028
- Difference in differences compared to control arm	−4.67 (−3.35, −5.91)	0.17 (−0.39, 0.81)	–		
Nepal					
- Baseline	4.7 (3.45, 6.25)	12.1 (10.04, 14.46)	5.6 (4.23, 7.27)	0.4930	0.2385
- End line	2.1 (1.30, 3.21)	6.0 (4.58, 7.72)	11.4 (9.40, 13.69)	0.0060	0.0366
- Difference in differences compared to control arm ^a^	−8.4 (−7.32, −9.46)	−11.9 (−10.43, −13.16)	–		

*Abbreviations*: *WL-RWSC* wall surface coverage with DWL up to 1.5 m in height from floor, *DWL-FWSC* wall surface coverage with DWL up to 1.8 m in height from floor, Control = no intervention

^a^Difference in differences compared to control arm = mean (B-A) – mean (D-C) Where, A = baseline count for the intervention group; B = post-intervention count for the intervention group; C = baseline count for the control group; D = post-intervention count for the control group. The difference in differences is zero if there are no changes of Female *P. argentipes* sand fly density after intervention. Negative sign represent reduction of Female *P. argentipes* sand fly count whereas positive sign represents increment

#### Adjusted intervention effect on sand fly density

Since the three study site clusters varied regarding baseline sand fly densities and other characteristics, we used a longitudinal regression model to adjust the effects of the variations between clusters. The results demonstrated that the effect of the DWL-RWSC and DWL-FWSC on sand fly density reduction remained significant both for the simple (unadjusted) and full (adjusted) model in pooled as well in stratified by sites analysis (Tables 4 and 5). The effect showed that about 73 and 63 % decrease of incidence rate of female *P. argentines* sand fly count attributed by the DWL-RWSC (IRR = 0.277, 95 % CI, 0.193, 0.397) and DWL-FWSC (IRR = 0.371, 95 % CI, 0.267, 0.514) respectively compared to control cluster. However, the difference of effect due to DWL-FWSC and DWL-RWSC intervention was not statistically significant (*p* = 0.214) (Table 4).

**Table 4:** Longitudinal regression analysis of pre-post control group design on pooled data

Time/Model	Parameter	IRR [95 % CI] (*p*-value)		*p*-value^*^
		DWL-RWSC	DWL-FWSC	
- Simple model	Unadjusted Intervention effect	0.277 [0.192, 0.397] (<0.0001)	0.367 [0.26, 0.510] (<0.0001)	0.214
- Full model	Adjusted Intervention effect	0.277 [0.193, 0.3971115] (<0.0001)^$1^	0.371 [0.267, 0.514] (<0.0001)^$2^	

*Abbreviations*: *WL-RWSC* wall surface coverage with DWL up to 1.5 m in height from floor, *DWL-FWSC* wall surface coverage with DWL up to 1.8 m in height from floor

^$1^Full model adjusted by the covariates: Family member slept at Varanda, HH knowledge about VL vector, Regular use of bed-net

^$2^Full model adjusted by the covariates: Family member slept at Varanda, Mud floor, # of bed-net less than 2 in house, House sprayed (IRS) in last 6 months

^*^ *p*-value for comparison of efficacy (Female *P. argentipes *sand fly density reduction) between DWL-RWSC and DWL-FWSC

**Table 5:** Longitudinal regression analysis of pre-post control group design on site specific data

Time/Model	Parameter	IRR [95 % CI] (*p*-value)	
		DWL-RWSC	DWL-FWSC
Bangladesh Site			
- Simple model	Unadjusted Intervention effect	0.476 [0.282, 0.801] (0.005)	0.432 [0.223, 0.836] (0.013)
− Full model	Adjusted Intervention effect	0.476 [0.282, 0.801] (0.005) ^a^	0.432 [0.223, 0.836] (0.013) ^b^
India Site			
- Simple model	Unadjusted Intervention effect	0.069 [0.016, 0.303] (<0.0001)	0.077 [0.010, 0.603] (<0.015)
- Full model	Adjusted Intervention effect	0.069 [0.016, 0.303] (<0.0001) ^c^	0.077 [0.010, 0.603] (<0.015) ^d^
Nepal Site			
- Simple model	Unadjusted Intervention effect	0.219 [0.120, 0.402] (<0.0001)	0.244 [0.156, 0.380] (<0.0001)
- Full model	Adjusted Intervention effect	0.219 [0.120, 0.402] (<0.0001) ^e^	0.244, [0.156, 0.380] (<0.0001) ^f^

*Abbreviations*: *DWL-RWSC* wall surface coverage with DWL up to 1.5 m in height from floor, *DWL-FWSC* wall surface coverage with DWL up to 1.8 m in height from floor

^a^Full model adjusted by the covariates: Family member slept at Varanda, HH asset score, HH knowledge about VL vector, Regular use of bed-net

^b^Full model adjusted by the covariates: HH asset score, Crack in wall, House sprayed in last 6 months

^c^Similar to crude regression model as none of the variables identified as confounders by the bivariae analysis

^d^Full model adjusted by the covariates: Family size > =5, # of bed-net <2 in house

^e^Similar to crude regression model as none of the variables identified as confounders by the bivariae analysis

^f^Full model adjusted by the covariates: Mud wall, HH asset score, Crack in wall, House sprayed in last 6 months

#### Sand fly mortality

At 1 month after intervention, the WHO cone bioassay test revealed an Abbot corrected mortality rate for the sand fly for DWL-FWSC of 92.23 % (95 % CI, 90.14 %–94.32 %) and for DWL-RWSC of 89.83 % (95 % CI, 87.73 %–91.94 %). In general the sand fly mortality rate was slightly less in India compare to Bangladesh and Nepal (Table 6).

**Table 6:** The Abbot-corrected sand fly mortality recorded in bio-assays on intervention surfaces in pooled as well as site specific data at 1-month after intervention

	Average corrected sand fly mortality (95 % CI)
Bangladesh	
- DWL-RWSC	90.70 % (87.00 %–94.38 %)
- DWL-FWSC	95.34 % (92.00 %–98.69 %)
India	
- DWL-RWSC	86.59 % (84.08 %–89.09 %)
- DWL-FWSC	85.81 % (83.60 %–88.01 %)
Nepal	
- DWL-RWSC	92.23 % (87.45 %–97.01 %)
- DWL-FWSC	95.54 % (94.88 %–96.21 %)
Overall/Pooled	
- DWL-RWSC	89.83 % (87.73 %–91.94 %)
- DWL-FWSC	92.23 % (90.14 %–94.32 %)

*Abbreviations*: *DWL-RWSC* wall surface coverage with DWL up to 1.5 m in height from floor, *DWL-FWSC* wall surface coverage with DWL up to 1.8 m in height from floor

### Cost, acceptability and adverse event

The household heads in intervention clusters expressed their high satisfaction for both DWL-FWSC and DWL-RWSC. The operation cost including materials and accessories in USD per household for DWL-RWSC and DWL-FWSC was respectively 17.75 and 20.76 (Table 7). The incremental cost-efficacy ratio was −6.36 (DWL-RWSC dominates DWL-FWSC). Sensitivity analysis showed that DWL-RWSC dominates DWL-FWSC in the majority of results (particularly in Bangladesh and India and also using pooled estimates; in Nepal most results lie on quadrant III of cost-efficacy plane, suggesting DWL-RWSC is mostly cost-saving, but also has lower efficacy). Thirty three percent of households reported adverse events which were mostly in India (see Additional file 1: Table S2). The most common adverse events were transient burning on the face followed by skin itching. Adverse events were more common with DWL-RWSC clusters compare to DWL-FWSC clusters (see Additional file 1: Table S2).

**Table 7:** Intervention cost

	Bangladesh	India	Nepal	Total
Meeting and training cost	147.0	2632.23	220.09	2999
Personnel cost (fixed staff during intervention month)	1471	695	331	2497.75
Cost of accessories related to intervention, [a]				
−DWL-RWSC	57.36	551.98	49.92	659.26
−DWL-FWSC	67.74	611.18	49.92	728.84
IDWL roll used, [b]				
−DWL-RWSC	5	3	1.5	9.5
−DWL-FWSC	6	5	2	13
Cost of IDWL, [c = b*50]				
−DWL-RWSC	250	150	75	475
−DWL-FWSC	300	250	100	650
Operational (staff travel and daily allowance) cost for intervention, [d]				
−DWL-RWSC	241.78	498.53	787.67	1527.98
−DWL-FWSC	302.23	532.97	787.67	1622.87
# of household under intervention [e]				
−DWL-RWSC	50	50	50	150
−DWL-FWSC	49	50	48	147
Operational cost per household (only staff and transportation cost); (d/e)				
−DWL-RWSC	4.84	9.97	15.75	10.19
−DWL-FWSC	6.17	10.66	16.41	11.04
Operation cost per HH including accessories cost; (a + d)/e				
−DWL-RWSC	5.98	21.01	16.75	14.58
−DWL-FWSC	7.55	22.88	17.45	16.00
Operational cost per HH including accessories and intervention material cost i.e., IDWL roll cost; (a + c + d)/e				
−DWL-RWSC	10.98	24.01	18.25	17.75
−DWL-FWSC	13.67	27.88	19.53	20.42
−Difference in intervention cost/HH (HL-FL) [f]	−2.69	−3.87	−1.28	−2.67
Efficacy (reduction on mean sand fly count/HH)				
−DWL-RWSC	2.80	4.67	8.40	5.38
−DWL-FWSC	1.10	−0.17	11.90	4.96
−Difference in efficacy (DWL-RWSC) [g]	1.70	4.84	−3.50	0.42
Incremental cost-efficacy ratio [f/g]	−1.58, DWL-RWSC dominates	−0.80, DWL-RWSC dominates	0.37, Quadrant III	−6.36, DWL-RWSC dominates
Proportion of results in sensitivity analysis where DWL-RWSC dominates	86.5 %	74.4 %	0 %	57.1 %
Proportion of results in sensitivity analysis where IDWL-FWSC is dominated	0 %	0 %	37.1 %	5.6 %

*Abbreviations*: *DWL-RWSC* wall surface coverage with DWL up to 1.5 m in height from floor, *DWL-FWSC* wall surface coverage with DWL up to 1.8 m in height from floor

## Discussion

Controlling sand fly levels to reduce the VL burden is crucial. Previously, VL disappeared due to the collateral benefit of the mosquito control program from 1960 to 1970 in the Indian sub-continent [4]. However, after ceasing IRS for mosquitoes, VL remerged to very high levels. This historical experience informs us that it is necessary to maintain sands fly densities under control to eventually succeed in the VL elimination program in the Indian sub-continent. The experience also informs us that when the disease burden goes down, the Government(s) becomes less inclined to maintain vector control due to its cost and organizational demands. When the VL case load goes down to a several hundred, the Government reallocates scarce resources to other health areas with more public health importance. This is why researchers and experts are desperately looking for safe, affordable and cost-effective methods for sand fly control, particularly for maintaining the success of the VL elimination program.

The DWL could be such a tool for long term control of the sand fly. In our previous study, we demonstrated its safety and efficacy for controlling sand fly in the Indian sub-continent through a randomized control trial [13]. In our earlier study we found 72 % reduction of sand fly density at 1 month follow up after intervention with DWL-FWSC [13]. In the current study, we determined that the similar efficacy regarding sand fly control can be achieved by reduced coverage of household wall surface with the DWL. DWL-RWSC contributed to 73 % reduction in sand fly density at 1 month. Sand fly density reduction by IRS and by commercial LLINs was 73 and 42 % at 5 months respectively [10]. Another study reported a reduction of sand fly density of 25 % at 12 months [15]. The impregnated existing bed nets with slow release insecticide tablets reduced sand fly density by about 65 % at 12 months [12]. These studies differ regarding study design, follow up time, methods for sand fly collection and analysis making those incomparable. But none of those investigated efficacy of DWL for sand fly control which we did in our previous study and in the current study [13].

In this study the efficacy of DWL-RWSC did not varied while analysis was done by crude and adjusted regression model expressing that the efficacy of the DWL-RWSC was independent of house type, deployed IRS and baseline sand fly density within clusters. The incremental cost-efficacy ratio of DWL-RWSC dominated over that of DWL-FWSC indication that DWL-RWSC substantially will reduce the DWL material cost and make installation easier.

We cannot compare the study results with others simply because studies with DWL for controlling sand fly do not exist except for our previous study [13]. When compared with our previous study, we did not find substantial difference in efficacy for sand fly control by reducing wall coverage with DWL at 1 month after intervention. The acceptability of reduced coverage with DWL was as high as with full coverage; however the adverse events were comparatively more common with DWL-RWSC. We cannot explain this. Fortunately all of those were transient and no serious adverse event was observed.

There are several limitations of the study. We could not explore the long term efficacy of the DWL-RWSC intervention. Future studies are needed to assess the longevity of efficacy by DWL-RWSC intervention. Another limitation is that we did not have an epidemiological end point which was due to the low incidence of VL in all three countries making this not feasible. It is important that a vector control method result in a reduction of the vector if it is to have an impact on the vector borne disease. The study did not include parasite infection in sand fly as an alternative way for impact evaluation. This was not possible due to lack of laboratory infrastructure, needed for this purpose. Nevertheless the achieved sand fly reduction of about 60–70 % by DWL-RWSC should contribute to VL elimination. For VL elimination in the Indian sub-continent the model-based estimated required *P. argentipes* sand fly density reduction was 67 % [16]. Our study demonstrated that DWL-RWSC was capable to reduce *P. argentipes* sand fly density with similar strength.

## Conclusions

We conclude that the evidence for controlling sand fly with DWL-RWSC is strong and should be considered by the VL elimination program in the Indian sub-continent.

## Additional file

Additional file 1: Table S1 of bivariate analysis of household related variables in the study area (site specific data). **Table S2** of adverse events related to intervention (DOCX 27 kb)

## Abbreviations

BPKIHS: B.P. Koirala Institute of Health Sciences
CDC: Centre For Disease Control
CI: Confidence interval
DWL: Durable Wall Lining
DWL-FWSC: Full wall surface coverage with 1.8 m DWL
DWL-RWSC: Reduced wall surface coverage with 1.5 m DWL
GEE: Generalized estimating equation
HH: Household
icddr,b: International Centre For Diarrhoeal Disease Research, Bangladesh
IRS: Indoor residual spraying with insecticides
RMRIMS: Rajendra Memorial Research Institute of Medical Sciences
SD: Standard deviation
TDR: Special programme for tropical disease research
VL: Visceral leishmaniasis
WHO: World Health Organization
