# Supplementary material for: Entomological efficacy of durable wall lining with reduced wall surface coverage for strengthening visceral leishmaniasis vector control in Bangladesh, India and Nepal
Source: BMC Infect Dis. 2016 Oct 6;16:539. doi: 10.1186/s12879-016-1881-8 (PMC5052807; doi:10.1186/s12879-016-1881-8)
Supplement: Additional file 1: Table S1 — of bivariate analysis of household related variables in the study area (site specific data). Table S2 of adverse events related to intervention (DOCX 27 kb) [file 12879_2016_1881_MOESM1_ESM.docx]

# Additional files

**Additional file 1 –Table of bivariate analysis of household related variables in the study area (site specific data)**

| **Study site** | **Control cluster, % (n)** | **Intervention clusters** | | | | **Total, % (n)** |
| --- | --- | --- | --- | --- | --- | --- |
|  |  | **DWL-RWSC, % (n)** | **P-value** | **DWL-FWSC, % (n)** | **P-value** |  |
| **Bangladesh** | N=10 | N=10 |  | N=10 |  | N=30 |
| Illiterate household head | 40.0 (4) | 20.0 (2) | 0.628 | 30.0 (3) | 1.000 | 30.0 (9) |
| Labor household head | 80.0 (8) | 70.0 (7) | 1.000 | 70.0 (7) | 1.000 | 73.3 (22) |
| Family size > =5 | 50.0 (5) | 20.0 (2) | 0.350 | 40.0 (4) | 1.000 | 36.7 (11) |
| Bed-rooms <2 | 70.0 (7) | 50.0 (5) | 0.650 | 80.0 (8) | 1.000 | 66.7 (20) |
| Family members slept at Varanda during the hot season | 100 (10) | 50.0 (5) | **0.033** | 100 (10) | -- | 83.3 (25) |
| Having cattle shed | 60.0 (6) | 80.0 (8) | 0.628 | 80.0 (8) | 0.628 | 73.3 (22) |
| Housing materials: |  |  |  |  |  |  |
| - Mud wall | 100 (10) | 100 (10) | -- | 100 (10) | -- | 100 (30) |
| - Mud floor | 100 (10) | 100 (10) | -- | 100 (10) | -- | 100 (30) |
| HH asset score |  |  |  |  |  |  |
| - Low | 100 (10) | 60.0 (6) | **0.087** | 10.0 (1) | **<0.0001** | 56.7 (17) |
| - Medium | 0.0 (0) | 0.0 (0) |  | 0.0 (0) |  | 0.0 (0) |
| - High | 0.0 (0) | 40.0 (4) |  | 90.0 (9) |  | 43.3 (13) |
| Crack in wall | 70.0 (7) | 70.0 (7) | 1.000 | 20.0 (2) | **0.070** | 53.3 (16) |
| Damp floor | 0.0 (0) | 0.0 (0) | -- | 0.0 (0) | -- | 0.0 (0) |
| HH head aware about VL | 100 (10) | 100 (10) | -- | 100 (10) | -- | 100 (30) |
| HH head aware about VL vector | 60.0 (6) | 0.0(0) | **0.011** | 40.0 (4) | 0.656 | 33.3 (10) |
| Having bed-net in house | 100 (10) | 100 (10) | -- | 100 (10) | -- | 100 (30) |
| # bed-net <2 in house | 30.0 (3) | 20.0 (2) | 1.000 | 0.0 (0) | 0.211 | 16.7 (5) |
| Regular use of bed-net | 20.0 (2) | 60.0 (6) | **0.170** | 10.0 (1) | 1.000 | 30.0 (9) |
| Other insecticides use: |  |  |  |  |  |  |
| - Mosquito coil | 20.0 (2) | 0.0 (0) | 0.474 | 0.0 (0) | 0.474 | 6.7 (2) |
| - Repellents | 0.0 (0) | 0.0 (0) | -- | 0.0 (0) | -- | 0.0 (0) |
| - Spray | 0.0 (0) | 0.0 (0) | -- | 0.0 (0) | -- | 0.0 (0) |
| - Smoke/dhup | 0.0 (0) | 0.0 (0) | -- | 0.0 (0) | -- | 0.0 (0) |
| - Others | 0.0 (0) | 0.0 (0) | -- | 0.0 (0) | -- | 0.0 (0) |
| House sprayed with insecticide (IRS) within last 6 months | 0.0 (0) | 0.0 (0) | -- | 100 (10) | **<0.0001** | 33.3 (10) |
| **India** | N=6 | N=6 |  | N=6 |  | N=18 |
| Illiterate household head | 83.3 (5) | 100 (6) | 1.000 | 33.3 (2) | 0.242 | 72.2 (13) |
| Labor household head | 100 (6) | 100 (6) | -- | 50.0 (3) | 0.182 | 83.3 (15) |
| Family size > =5 | 16.7 (1) | 33.3 (2) | 1.000 | 83.3 (5) | **0.080** | 44.4 (8) |
| Bed-rooms <2 | 66.7 (4) | 83.3 (5) | 1.000 | 16.7 (1) | 0.242 | 55.6 (10) |
| Family members slept at Varanda during the hot season | 16.7 (1) | 16.7 (1) | 1.000 | 66.7 (4) | 0.242 | 33.3 (6) |
| Having cattle shed | 16.7 (1) | 16.7 (1) | 1.000 | 16.7 (1) | 1.000 | 16.7 (3) |
| Housing materials: |  |  |  |  |  |  |
| - Mud wall | 33.3 (2) | 83.3 (5) | 0.242 | 16.7 (1) | 1.000 | 44.4 (8) |
| - Mud floor | 100 (6) | 83.3 (5) | 1.000 | 66.7 (4) | 0.455 | 83.3 (15) |
| HH asset score |  |  |  |  |  |  |
| - Low | 100 (6) | 100 (6) | **--** | 83.3 (5) | 1.000 | 94.4 (17) |
| - Medium | 0.0 (0) | 0.0 (0) |  | 0.0 (0) |  | 0.0 (0) |
| - High | 0.0 (0) | 0.0 (0) |  | 16.7 (1) |  | 5.6 (1) |
| Crack in wall | 100 (6) | 100 (6) | -- | 66.7 (4) | 0.455 | 88.9 (16) |
| Damp floor | 0.0 (0) | 0.0 (0) | -- | 0.0 (0) | -- | 0.0 (0) |
| HH head aware about VL | 100 (6) | 100 (6) | -- | 100 (6) | -- | 100 (18) |
| HH head aware about VL vector | 0.0 (0) | 0.0 (0) | -- | 0.0 (0) | -- | 0.0 (0) |
| Having bed-net in house | 16.7 (1) | 33.3 (2) | 1.000 | 66.7 (4) | 0.242 | 38.9 (7) |
| # bed-net <2 in house | 100 (6) | 100 (6) | -- | 33.3 (2) | **0.061** | 77.8 (14) |
| Regular use of bed-net | 0.0 (0) | 0.0 (0) | -- | 0.0 (0) | -- | 0.0 (0) |
| Other insecticides use: |  |  |  |  |  |  |
| - Mosquito coil | 0.0 (0) | 0.0 (0) | -- | 0.0 (0) | -- | 0.0 (0) |
| - Repellents | 0.0 (0) | 0.0 (0) | -- | 0.0 (0) | -- | 0.0 (0) |
| - Spray | 0.0 (0) | 0.0 (0) | -- | 0.0 (0) | -- | 0.0 (0) |
| - Smoke/dhup | 100 (6) | 100 (6) | -- | 100 (6) | -- | 100 (18) |
| - Others | 0.0 (0) | 0.0 (0) | -- | 0.0 (0) | -- | 0.0 (0) |
| House sprayed with insecticide (IRS) within last 6 months | 0.0 (0) | 0.0 (0) | -- | 0.0 (0) | -- | 0.0 (0) |
| **Nepal** | N=10 | N=10 |  | N=10 |  | N=30 |
| Illiterate household head | 10.0 (1) | 30.0 (3) | 0.582 | 0.0 (0) | 1.000 | 13.3 (4) |
| Labor household head | 50.0 (5) | 60.0 (6) | 1.000 | 30.0 (3) | 0.650 | 46.7 (14) |
| Family size > =5 | 90.0 (9) | 60.0 (6) | 0.303 | 70.0 (7) | 0.582 | 73.3 (22) |
| Bed-rooms <2 | 10.0 (1) | 40.0 (4) | 0.303 | 20.0 (2) | 1.000 | 23.3 (7) |
| Family members slept at Varanda during the hot season | 60.0 (6) | 50.0 (5) | 1.000 | 90.0 (9) | 0.303 | 66.7 (20) |
| Having cattle shed | 100 (10) | 90.0 (9) | 1.000 | 90.0 (9) | 1.000 | 93.3 (28) |
| Housing materials: |  |  |  |  |  |  |
| - Mud wall | 100 (10) | 90.0 (9) | 1.000 | 60.0 (6) | **0.087** | 83.3 (25) |
| - Mud floor | 100 (10) | 90.0 (9) | 1.000 | 70.0 (7) | 0.211 | 86.7 (26) |
| HH asset score |  |  |  |  |  |  |
| - Low | 0.0 (0) | 10.0 (1) | 1.000 | 40.0 (4) | **0.087** | 16.7 (5) |
| - Medium | 0.0 (0) | 0.0 (0) |  | 0.0 (0) |  | 0.0 (0) |
| - High | 100 (10) | 90.0 (9) |  | 60.0 (6) |  | 83.3 (25) |
| Crack in wall | 40.0 (4) | 40.0 (4) | 1.000 | 80.0 (8) | **0.170** | 53.3 (16) |
| Damp floor | 0.0 (0) | 10.0 (1) | 1.000 | 0.0 (0) | **--** | 3.3 (1) |
| HH head aware about VL | 90.0 (9) | 90.0 (9) | 1.000 | 50.0 (5) | 0.141 | 76.7 (23) |
| HH head aware about VL vector | 10.0 (1) | 0.0 (0) | 1.000 | 10.0(1) | 1.000 | 6.7 (2) |
| Having bed-net in house | 100 (10) | 90.0 (9) | 1.000 | 90.0 (9) | 1.000 | 93.3 (28) |
| # bed-net <2 in house | 10.0 (1) | 30.0 (3) | 0.582 | 10.0 (1) | 1.000 | 16.7 (5) |
| Regular use of bed-net | 80.0 (8) | 80.0 (8) | 1.000 | 80.0 (8) | 1.000 | 80.0 (24) |
| Other insecticides use: |  |  |  |  |  |  |
| - Mosquito coil | 30.0 (3) | 10.0 (1) | 0.582 | 30.0 (3) | 1.000 | 23.3 (7) |
| - Repellents | 0.0 (0) | 0.0 (0) | -- | 0.0 (0) | -- | 0.0 (0) |
| - Spray | 10.0 (1) | 0.0 (0) | 1.000 | 0.0 (0) | 1.000 | 3.3 (1) |
| - Smoke/dhup | 10.0 (1) | 0.0 (0) | 1.000 | 0.0 (0) | 1.000 | 3.3 (1) |
| - Others | 0.0 (0) | 0.0 (0) | -- | 0.0 (0) | -- | 0.0 (0) |
| House sprayed with insecticide (IRS) within last 6 months | 0.0 (0) | 10.0 (1) | 1.000 | 0.0 (0) | -- | 3.3 (1) |

**Additional file 2 –Table of adverse events related to intervention**

|  | **Bangladesh,** % (n/N) | **India,** %(n/N) | **Nepal,** %(n/N) | **Overall,** %(n/N) |
| --- | --- | --- | --- | --- |
| Any unusual events |  |  |  |  |
| - DWL-RWSC | 0.0 (0/50) | 0.0 (0/50) | 8.5 (4/47) | 2.7 (4/147) |
| - DWL-FWSC | 2.0 (1/49) | 0.0 (0/50) | 33.3 (14/42) | 10.6 (15/141) |
| Experienced symptoms |  |  |  |  |
| Coughing |  |  |  |  |
| - DWL-RWSC | 0.0 (0/50) | 0.0 (0/50) | 2.1 (1/47) | 0.7 (1/147) |
| - DWL-FWSC | 0.0 (0/49) | 0.0 (0/50) | 2.4 (1/42) | 0.7 (1/141) |
| Dizziness |  |  |  |  |
| - DWL-RWSC | 0.0 (0/50) | 0.0 (0/50) | 0.0 (0/47) | 0.0 (0/147) |
| - DWL-FWSC | 2.0 (1/49) | 0.0 (0/50) | 0.0 (0/42) | 0.7 (1/141) |
| Fever |  |  |  |  |
| - DWL-RWSC | 0.0 (0/50) | 0.0 (0/50) | 0.0 (0/47) | 0.0 (0/147) |
| - DWL-FWSC | 0.0 (0/49) | 0.0(0/50) | 4.8 (2/42) | 1.4 (2/141) |
| Headache |  |  |  |  |
| - DWL-RWSC | 0.0 (0/50) | 0.0 (0/50) | 0.0 (0/47) | 0.0 (0/147) |
| - DWL-FWSC | 0.0 (0/49) | 0.0 (0/50) | 9.5 (4/42) | 2.8 (4/141) |
| Itching |  |  |  |  |
| - DWL-RWSC | 0.0 (0/50) | 68.0 (34/50) | 10.6 (5/47) | 26.5 (39/147) |
| - DWL-FWSC | 2.0 (1/49) | 46.0 (23/50) | 14.3 (6/42) | 21.3 (30/141) |
| Running nose |  |  |  |  |
| - DWL-RWSC | 0.0 (0/50) | 0.0 (0/50) | 0.0 (0/47) | 0.0 (0/147) |
| - DWL-FWSC | 0.0 (0/49) | 0.0 (0/50) | 2.4 (1/42) | 0.7 (1/141) |
| Sore eyes |  |  |  |  |
| - DWL-RWSC | 0.0 (0/50) | 0.0 (0/50) | 0.0 (0/47) | 0.0 (0/147) |
| - DWL-FWSC | 4.1 (2/49) | 0.0 (0/50) | 0.0 (0/42) | 1.4 (2/141) |
| Burning sensation on face |  |  |  |  |
| - DWL-RWSC | 0.0 (0/50) | 56.0 (28/50) | 2.1 (1/47) | 19.7 (29/147) |
| - DWL-FWSC | 4.1 (2/49) | 78.0 (39/50) | 7.1 (3/42) | 31.2 (44/141) |
| Sleeplessness |  |  |  |  |
| - DWL-RWSC | 0.0 (0/50) | 0.0 (0/50) | 0.0 (0/47) | 0.0 (0/147) |
| - DWL-FWSC | 0.0 (0/49) | 0.0 (0/50) | 0.0 (0/42) | 0.0 (0/141) |
| Sneezing |  |  |  |  |
| - DWL-RWSC | 0.0 (0/50) | 0.0 (0/50) | 0.0 (0/47) | 0.0 (0/147) |
| - DWL-FWSC | 0.0 (0/49) | 0.0 (0/50) | 11.9 (5/42) | 3.5 (5/141) |
| Stomach ache |  |  |  |  |
| - DWL-RWSC | 0.0 (0/50) | 0.0 (0/50) | 0.0 (0/47) | 0.0 (0/147) |
| - DWL-FWSC | 0.0 (0/49) | 0.0 (0/50) | 0.0 (0/42) | 0.0 (0/141) |
| Unpleasant smell |  |  |  |  |
| - DWL-RWSC | 0.0 (0/50) | 0.0 (0/50) | 0.0 (0/47) | 0.0 (0/147) |
| - DWL-FWSC | 0.0 (0/49) | 0.0 (0/50) | 0.0 (0/42) | 0.0 (0/141) |
| Unpleasant taste |  |  |  |  |
| - DWL-RWSC | 0.0 (0/50) | 0.0 (0/50) | 0.0 (0/47) | 0.0 (0/147) |
| - DWL-FWSC | 0.0 (0/49) | 0.0 (0/50) | 0.0 (0/42) | 0.0 (0/141) |
| Others |  |  |  |  |
| - DWL-RWSC | 4.0 (2/50) | 0.0 (0/50) | 0.0 (0/47) | 1.4 (2/147) |
| - DWL-FWSC | 30.6 (15/49) | 0.0 (0/50) | 0.0 (0/42) | 10.6 (15/141) |

*DWL-RWSC: wall surface coverage with DWL up to 1.0 m in height from floor, DWL-FWSC: wall surface coverage with DWL up to 1.8 m in height from floor,*
